# Toward an interoperable, intraoperative situation recognition system via process modeling, execution, and control using the standards BPMN and CMMN

**DOI:** 10.1007/s11548-023-03004-y

**Published:** 2023-08-24

**Authors:** Denise Junger, Elisaveta Just, Johanna M. Brandenburg, Martin Wagner, Katharina Schaumann, Thomas Klenzner, Oliver Burgert

**Affiliations:** 1 School of Informatics, Research Group Computer Assisted Medicine (CaMed), Reutlingen University, Reutlingen, Germany; 2 Department of General, Visceral and Transplantation Surgery, Heidelberg University Hospital, Heidelberg, Germany; 3 National Center for Tumor Diseases Heidelberg, Heidelberg, Germany; 4 Center for the Tactile Internet With Human in the Loop (CeTI), Technical University Dresden, Dresden, Germany; 5 Department of Otorhinolaryngology, University Hospital Düsseldorf, Düsseldorf, Germany

**Keywords:** Situation recognition, Surgical process modeling, BPMN, CMMN, Intraoperative area

## Abstract

**Purpose:**

For the modeling, execution, and control of complex, non-standardized intraoperative processes, a modeling language is needed that reflects the variability of interventions. As the established Business Process Model and Notation (BPMN) reaches its limits in terms of flexibility, the Case Management Model and Notation (CMMN) was considered as it addresses weakly structured processes.

**Methods:**

To analyze the suitability of the modeling languages, BPMN and CMMN models of a Robot-Assisted Minimally Invasive Esophagectomy and Cochlea Implantation were derived and integrated into a situation recognition workflow. Test cases were used to contrast the differences and compare the advantages and disadvantages of the models concerning modeling, execution, and control. Furthermore, the impact on transferability was investigated.

**Results:**

Compared to BPMN, CMMN allows flexibility for modeling intraoperative processes while remaining understandable. Although more effort and process knowledge are needed for execution and control within a situation recognition system, CMMN enables better transferability of the models and therefore the system. Concluding, CMMN should be chosen as a supplement to BPMN for flexible process parts that can only be covered insufficiently by BPMN, or otherwise as a replacement for the entire process.

**Conclusion:**

CMMN offers the flexibility for variable, weakly structured process parts, and is thus suitable for surgical interventions. A combination of both notations could allow optimal use of their advantages and support the transferability of the situation recognition system.

## Introduction

Formal modeling of surgical processes is one of the main aspects to achieve situation awareness in the operating room (OR) [1]. Surgical Process Models (SPM) are used to better understand and analyze surgical workflows [2] and can be applied for situation recognition purposes [3] to incorporate process knowledge into the recognition logic [4]. Formalization for automated handling and processing is therefore necessary, using, e.g., ontologies, XML schemas, or graphs [2]. To represent knowledge, several approaches state to use SPMs in general [5–8] or an ontology [5, 8–12], as analyzed in [3]. Several research groups rely on using the Business Process Model and Notation (BPMN) for modeling the course of an intervention [4, 13, 14]. BPMN models enable a graph-based visualization based on an underlying XML structure to provide machine readability and can be managed via a workflow engine. However, for modeling more complex and variable surgical workflows, BPMN reaches its limits, as activities within the OR can occur in variable order or be performed discretionarily. BPMN provides less flexibility [15] and becomes complex depicting process variants [16]. A modeling language that can reflect the variability of complex and highly flexible surgical workflows is crucial.

Case Management Model and Notation (CMMN) is a modeling language, promising to be more flexible than BPMN [17], and could therefore be well suited for modeling variable surgical interventions [18]. While Zensen and Küster [17] elaborated general advantages and disadvantages of BPMN and CMMN, the work of Wiemuth et al. [18] showed for the medical field how complex sub processes of a Cataract Operation are when modeled with BPMN and how the same process can be modeled easier in CMMN and Decision Model and Notation (DMN). The results showed that using CMMN and DMN to model flexible and weakly structured processes can lead to a more compact model while also being better readable. Furthermore, a combination of BPMN, CMMN, and DMN was addressed to depict a mixture of structured and unstructured process parts including decision support. Nevertheless, CMMN may be more difficult to understand than BPMN. The idea of [18] was further specified in [19] which showed a concept of situation recognition using a combination of BPMN, CMMN, and DMN for process modeling. A combination of BPMN and CMMN is also used in other domains, where it is described as a hybrid modeling approach that defines a process more efficiently while simplifying it [20] or a connection for integrating structured and loosely specified processes [15].

Another work using CMMN for modeling perioperative processes dealt with the transferability of SPMs [21]. Herzberg et al. [21] remodeled a BPMN model of an organ transplantation. To transfer the model to other hospitals, it should represent variable processes. This was achieved using CMMN, which enabled the modeling of the surgical intervention for multiple hospitals, including different treatments. Tasks that occur in all hospitals were modeled as required whereas non-obligatory tasks were depicted as discretionary. Moreover, it was discussed that realizing the same concept in BPMN would be harder, as the different variants need to be modeled via gateways, increasing the complexity of the model.

Concluding from [18] and [21], CMMN is promising to be more suitable for variable surgical interventions than BPMN. It allows tasks to be executed in variable order [22] and is fitting for the representation of optional steps [17]. As the work of [18] focused on theoretical analysis, it lacked a practical evaluation for the use case of situation recognition, also including the execution and control of the models. Furthermore, no CMMN without BPMN and DMN was demonstrated. The concept of [21] using CMMN for better transferability to other hospitals is another important aspect that should be discussed concerning situation recognition.

The Situation Recognition System (SRS) of Junger et al. [4] aims for a flexible and intervention-independent situation recognition in the OR. Knowledge from BPMN models is used in combination with sensor data to recognize the actual situation. Furthermore, the model is executed for workflow control. The gathered information about the situation in the OR is provided to extern context-aware systems independent of their usage (e.g., for automatic information filtering [11] or provision [8, 23, 24], device control [25, 26]). To demonstrate that the SRS can be adapted to support non-standardized interventions, more complex interventions should be integrated. Since BPMN can become quite complex [16, 20], CMMN should be considered as it is promising to keep the models compact for readability, while still depicting variability and optimizing the transferability of the SRS.

In this work, practical approaches of process modeling, execution, and control of more complex, non-standardized surgical interventions for situation recognition are analyzed. BPMN, CMMN, and combination models are developed for two interventions, the Robot-Assisted Minimally Invasive Esophagectomy (RAMIE) and Cochlea Implantation (CI). The established models are used within the extended SRS of [4] for accessing process knowledge and reflecting the course of the intervention. The evaluation comprises the differences in modeling, execution, and control of the SPMs, analyzing the modeling approaches and effects on the SRS workflow. The discussion elaborates on the advantages and disadvantages of the approaches and highlights the effect on the transferability of the SRS employing the models.

## Methods

### Surgical process modeling of the use cases robot-assisted minimally invasive esophagectomy and cochlea implantation

As the main use case, the RAMIE was chosen, being a complex and demanding intervention in visceral surgery [27] and a current case for phase recognition [28]. It comprises an abdominal part with gastric and distal esophageal mobilization, construction of a gastric tube, and lymphadenectomy along major abdominal vessels, as well as a thoracic part with dissection of the esophagus with lymphadenectomy, stomach pull-up, and an anastomosis of the remaining esophagus and gastric tube [29]. At multiple parts, steps can occur in variable order or be optional. Furthermore, several foreseen events are known. The SPM of [27] depicts the surgical workflow of the RAMIE comprising nine phases and various steps modeled in BPMN and was created as part of preliminary work with the clinical partners of Heidelberg University Hospital providing information like steps, variances, or used instruments. Because the model was intended to be used for an intraoperative checklist [30] that allows the flexible tick-off of the displayed steps, these were modeled as *Tasks* after each other using *Sequence Flows*. For the idea of a transferable SRS, a higher degree of variability is needed to cover the different possible courses of the intervention. Therefore, the SPM was extended to depict the variable order of steps and optional tasks.

**Fig. 1:**
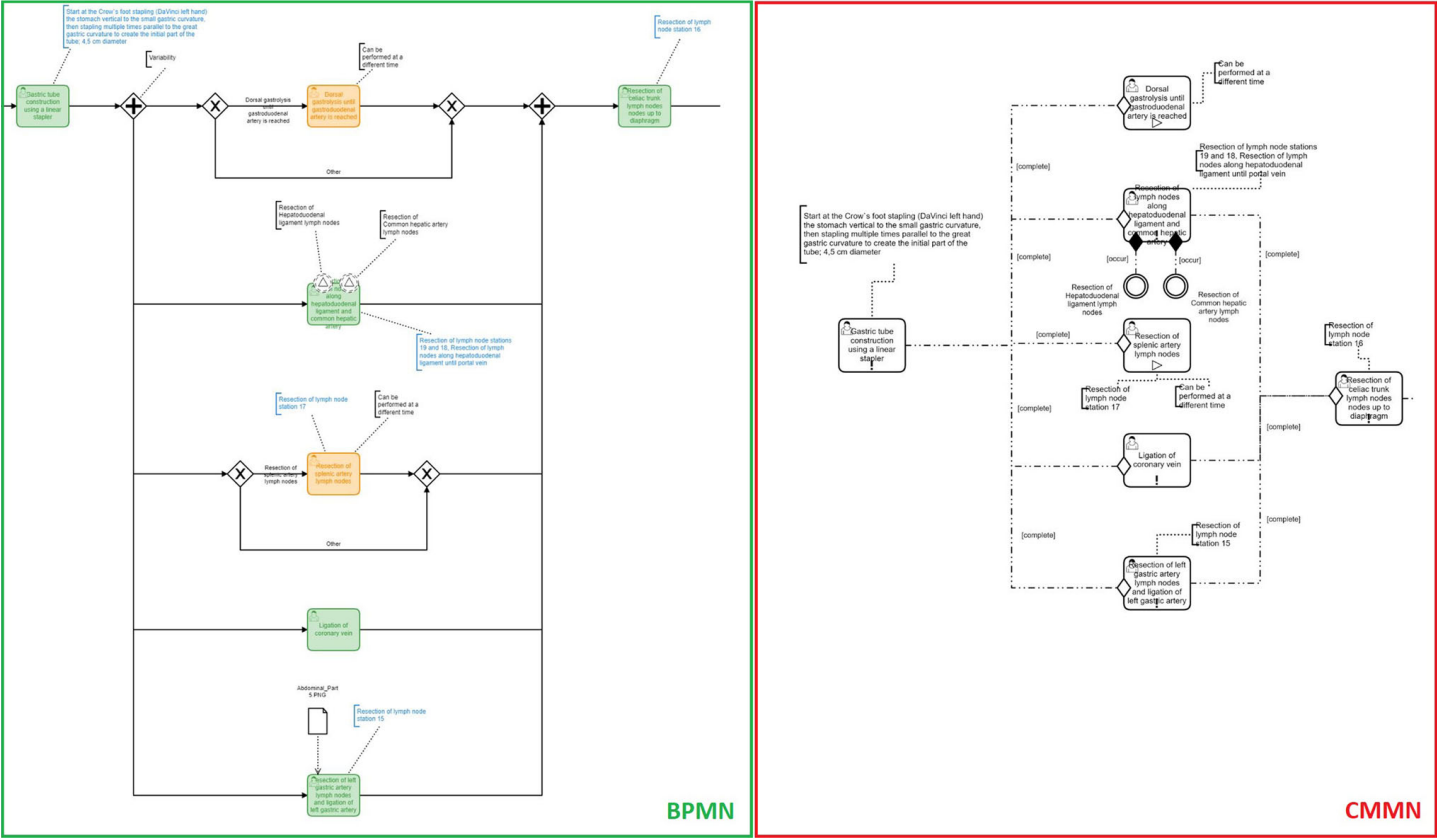
Excerpt of the RAMIE use case contrasting variable process parts modeled in BPMN (left) and CMMN (right), including steps in variable order and optional tasks

To allow process control, the modeled *Tasks* were switched to *User Tasks*. For the first version, *Exclusive (XOR) Gateways* were used for workflow parts where the execution order can vary. Due to the model becoming too complex (e.g., a process part with five tasks in variable order including optional tasks led to ~ 150 possible paths), it was rejected. After considering *Ad-hoc Sub Processes*, but these did not allow the desired distinction between mandatory and optional tasks, they were not used either. The new approach modeled variable steps using *Parallel (AND) Gateways* and optional steps using *XOR Gateways*. For a two-granularity (2G) version, the *User Tasks* (steps) were grouped into *Sub Processes* (phases). Events were exemplarily included using *Signal Boundary Events*. An excerpt of the BPMN model is depicted in Fig. 1 (left).

The very same intervention was realized using CMMN, based on the same clinical information. Steps were modeled as *Human Tasks*. *Sentries* and *Stages* were used to depict dependencies. Optional tasks were integrated without a *Required Rule* but a *Manual Activation Rule* inspired by [17], as *Discretionary Tasks* used in [21] were not supported by the Camunda Engine [31]. Events were exemplarily included using *Event Listener*. For the 2G version, *Stages* equivalent to the *Sub Processes* of BPMN were integrated. An excerpt of the CMMN model is depicted in Fig. 1 (right).

In addition, combination models comprising both notations were modeled. The “mixed” combination relies on a BPMN including *Call Activities* to CMMNs just for variable processes, based on [18]. The “structured” combination contains high-level *Stages* in BPMN and *Call Activities* referring to CMMNs including structured and variable steps. This approach was partly inspired by [19]. An overview of the used notation elements is depicted in Table 1.

**Table 1:** Overview of our transfer concept of process information to BPMN and CMMN elements

Process information	BPMN elements	CMMN elements
Phases and steps	*Sub Process* containing *User Tasks*	*Stage* containing *Human Tasks*
Order/Dependency	*Sequence Flow*	*Sentry*
Variable step sequence	*Exclusive (XOR) Gateway* (decision) & *Parallel (AND) Gateway* (variability)	If no order is defined
Required vs. optional steps	Required: No other path is possible Optional: Another path is possible	*Required Rule* & *Manual Activation Rule*
Foreseen events and the following measure	*Signal Boundary Event* followed by a step	*Event Listener* or optional step
Link from BPMN to CMMN	*Call Activity*	No reference used

As a second use case, the CI based on the BPMN model of [30], comprising steps structured in the five phases preparation, access, operation under and after the microscope, as well as follow-up, was used. This use case is more straightforward, less containing steps in variable order but includes different procedures, e.g., deviated steps according to the cochlea implant that is implanted, and foreseen events. For the BPMN, the model of [30] was used which was created as part of preliminary work with the clinical partners of University Hospital Düsseldorf. Different paths were realized by *XOR Gateways*, *Sub Processes* were used to structure steps in phases. From the BPMN model, a CMMN model was derived the same as for RAMIE, using *Sentries* and *Stages* to depict dependencies as well as phases and *Manual Activation Rules* to model different paths. Furthermore, a mixed and structured combination model was derived.

### Workflow within the situation recognition system using surgical process models

The RAMIE use case was integrated into the framework prototype of [4], including parameter combinations and situation rules. To simulate the intervention, acquired knowledge from Heidelberg University Hospital, provided by the clinical partners (see previous subsection), was used, concretely which instrument combination may be used and where the surgeon and assistant will probably be in the room in the respective steps. Therefore, reasonable sensor data combinations were simulated including the name of the used instruments and/or position data of the actors. In addition, the CI use case was integrated. As no sensor data were available for data simulation, we simulated the step name as input for situation recognition. Furthermore, the logic to support more complex BPMN models was extended. Control logic for the established CMMN and combination models were pre-tested in a separate test environment, simulating the step names via the checklist of [30]. The ticked-off checklist point, indicating the end of the respective situation, was used to control the process. Overall, 18 different models of RAMIE and 14 of CI, including CMMN cases of combination models, were tested. After pre-testing, parts of the logic were included, adapted, and extended to fit into the SRS architecture and functionality.

**Fig. 2:**
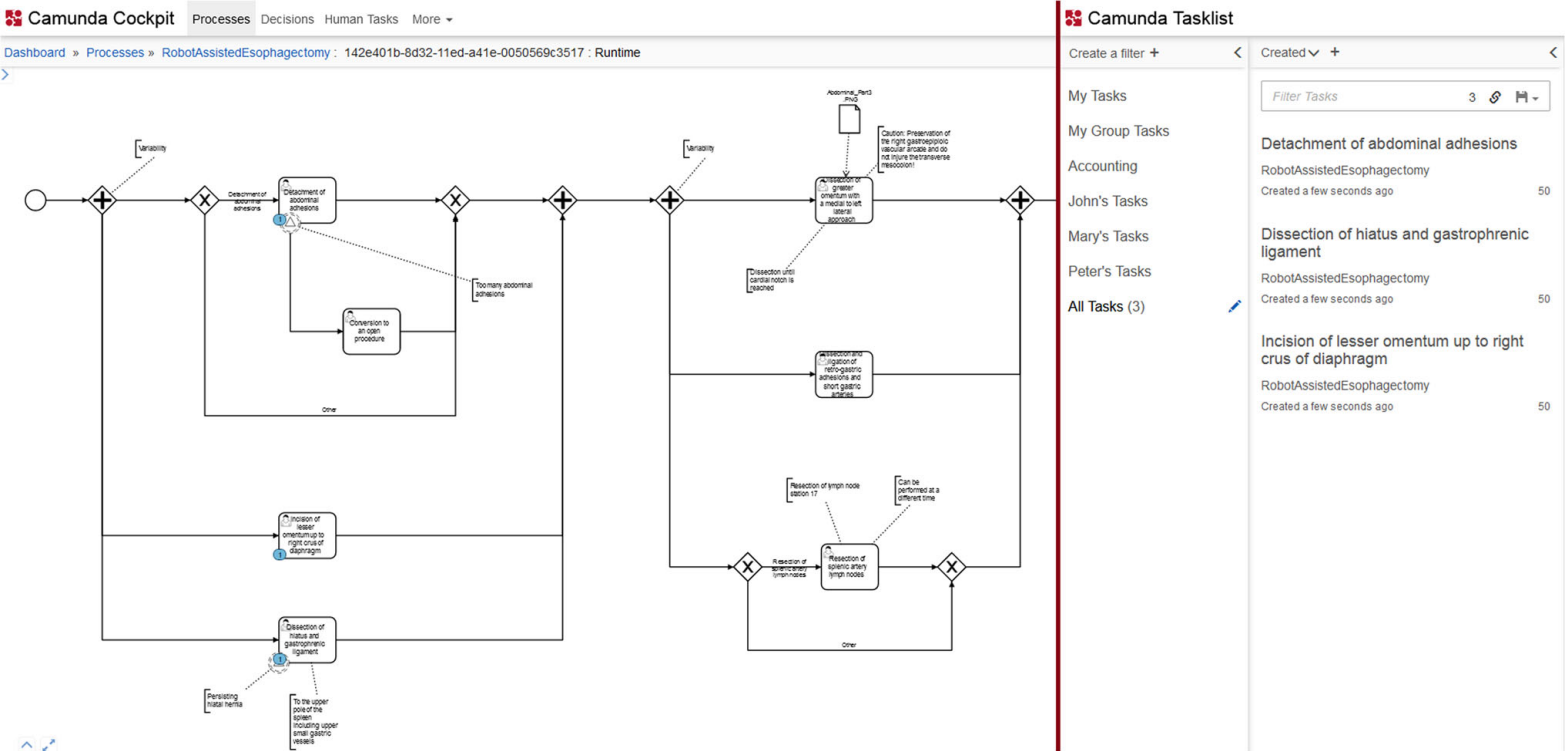
Camunda cockpit (left), visualizing the process token within the running process, and Camunda Tasklist (right), listing running activity instances of the process

In the following, the SRS functionality is depicted, concentrating on the adapted parts of the system. Further information on the overall concept can be retrieved from [4]. SPMs are used within the architecture for both, obtaining process knowledge and reflecting the actual course of the intervention (process control). The administration and control of the running processes are supported by a Camunda Engine [31] (see Fig. 2, left), whereby most of the control logic, unless otherwise specified, is implemented by the SRS itself. The SRS communicates with the engine via a middleware that provides REST API [32] functions, e.g., to complete a task.

#### Situation recognition

**Fig. 3:**
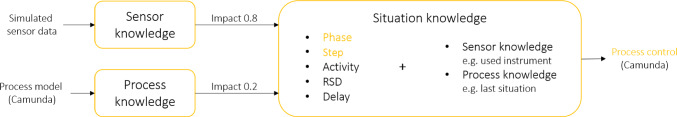
Combination of knowledge from simulated sensor data and the process model to form *Situation Knowledge* for process control via recognized phase and/or step, while *Impact* represents a configurable value that defines the factor of the *Sensor Knowledge* (here: 80%) and *Process Knowledge* (here: 20%) to form *Situation Knowledge*

For situation recognition, *Sensor Knowledge* (e.g., used instrument) retrieved from sensor data and *Process Knowledge* (e.g., next possible steps) retrieved from SPMs are used. Within modules for phase, step, and activity recognition, first *Situation Knowledge* (e.g., surgical step) is interpreted based on solely the *Sensor Knowledge* by using situation rules. Secondly, the resulting *Situation Knowledge* is enriched by *Process Knowledge* including the next possible situations. Hereby, a configured value defines the influence of the *Process Knowledge* (see Fig. 3). The module for calculating the remaining surgery duration (RSD) and delay only uses *Process Knowledge* of the duration.

#### Workflow management (get process knowledge)

To provide *Process Knowledge*, the XML of the intervention’s model is used. As the long-term concept is to strengthen the *Process Knowledge* by using similar, already executed case-relevant processes, this is temporarily simulated by always using the BPMN model of the intervention, since it reflects the expected input of similar cases in a structured manner. Via recursive function calls, valuable next steps are retrieved from the XML. Starting from the current step, the next steps are determined based on the modeled sequence by going through all possible paths.

#### Workflow management (process situation knowledge)

The process is controlled by the recognized situation with the highest probability. Recursive function calls are used to identify whether it is logical to start the next task by parsing the XML of the belonging intervention model. A list of instances that can be completed is constructed while comparing the previous tasks with actual running activity instances. Moreover, the information on whether the new situation is reasonable is returned. Afterward, the matching running task is completed in Camunda, so that the next possible *User/Human Tasks* are activated automatically.

### Evaluation

Comprehensibility of the process models is important for clinically correct models. Therefore, clinicians need to comprehend the models to give feedback or even create models themselves. To gain an impression and rough trend of the comprehensibility of the established RAMIE and CI models, a total of 12 user tests were conducted with clinicians associated with the University Hospitals of Heidelberg and Düsseldorf. For the evaluation, a simple form was used. First, it was noted if the modeling notations and intervention are familiar. After a small introduction to BPMN and CMMN, the established 2G BPMN, CMMN, and combination models were presented. The user was asked to explain the modeled process and to identify specific elements, like required or optional steps. The subjective impressions were recorded within the form. Via post-test questions, the user rated the comprehensibility of each model on a scale from 1 to 5 (very easy to very hard). Furthermore, the subjective opinion of the most comprehensible model was accessed. During the whole evaluation, remarks from the user and impressions of the evaluator were gathered.

On basis of the user tests, a selection of the established models was integrated into the SRS. For this purpose, minor changes, mostly referring to the removal of sub stages, were made to allow for comparable cases and uniform modeling. In the end, the 2G BPMN models, the adjusted 2G CMMN models, and the adjusted 2G structured models with a total of nine cases for RAMIE and five for CI were deployed. Although only a selection of models was integrated, the pre-test has already proven that all models can be executed and controlled accordingly.

To evaluate the SRS workflow with more complex interventions, sensor data were simulated via an automatic data simulation, defining a valid path through the process. Furthermore, the flexibility and error-proneness of the models and control logic were tested by simulating the steps of the intervention in unreasonable order via distorting the input data. The performed test cases cover the variable execution of tasks and different paths, the skipping of required and optional tasks, as well as the regression and repetition of tasks. The console output was used to comprehend the recognition steps, interpreted sensor, process, and situation knowledge, as well as subsequent control of the respective process model. In addition, the Camunda Cockpit and Tasklist (see Fig. 2) were viewed to follow the process via token and to retrace active tasks. Via the Camunda REST API, the correct execution of the models was retrieved. Modeled event information (e.g., bleeding) was not included in the tests for now. The system tests focus on the analysis of the execution and control of the process models like peculiarities in the execution and limitations due to the models. Therefore, the results are meant to highlight the recognition behavior in supporting different modeling approaches.

## Results

### Comparison of the surgical process models and user tests (modeling)

**Fig. 4:**
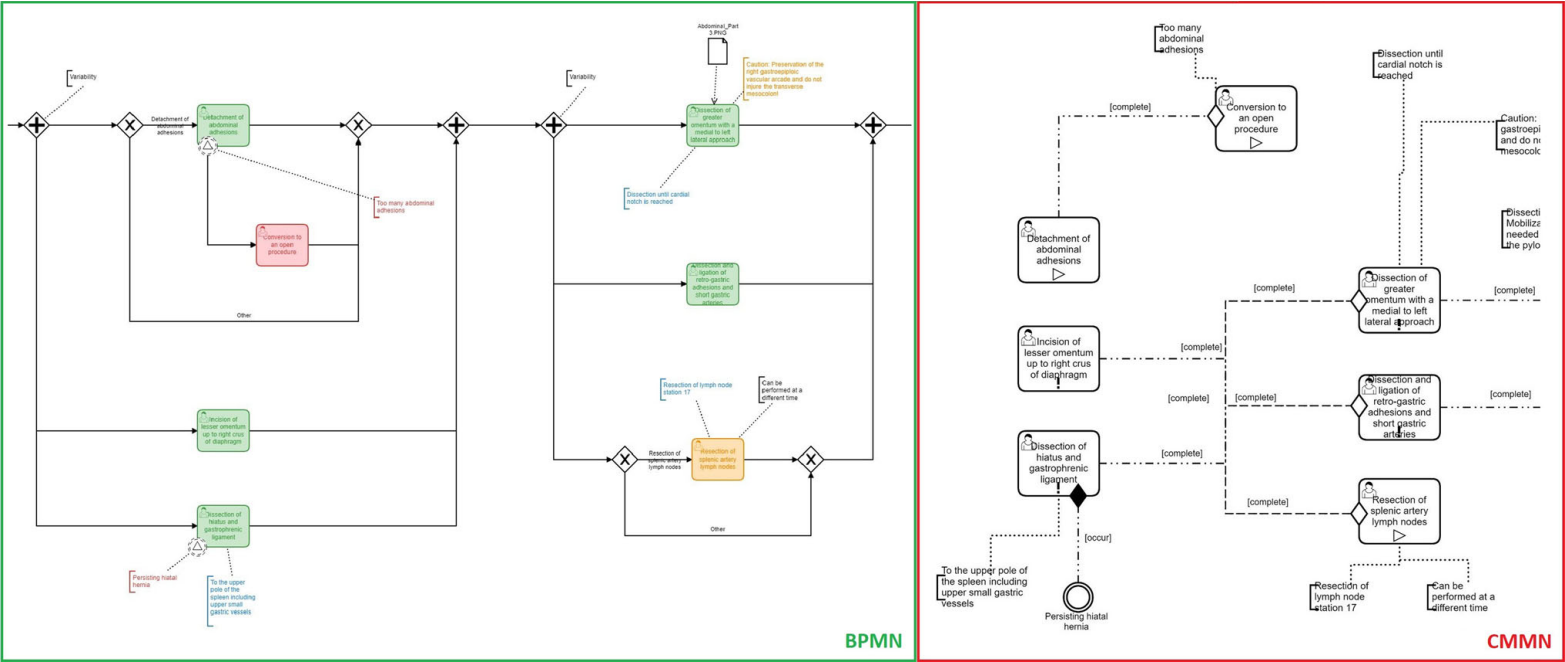
Excerpt of the RAMIE use case contrasting variable process parts modeled in BPMN (left) and CMMN (right), including steps in variable order and optional tasks

The RAMIE use case shows that via *Sequence Flows,* gateways, and start/end events a clear path is recognizable in BPMN (see Fig. 4, left). Optional tasks can be identified via *XOR Gateways*. Nevertheless, tasks in variable order need to be modeled via workaround (*AND Gateways*), intended to be executed after each other, thus indirectly semi-parallel and not correctly depicted. In contrast, tasks in CMMN can be modeled with fewer dependencies without gateways or start/end events but also *Sentries* and *Stages*, making it possible to represent variable sequences while maintaining some structure (see Fig. 4, right). Via *Sentries* as *Entry Criteria*, sequences to following tasks can be depicted without clarifying their order. The tasks with the *Entry Criteria* are dependent on the completion of the previous elements (“completed” event). Required and optional tasks can simply be recognized via the symbols within the modeled tasks (*Required Rule* and *Manual Activation Rule*).

**Fig. 5:**
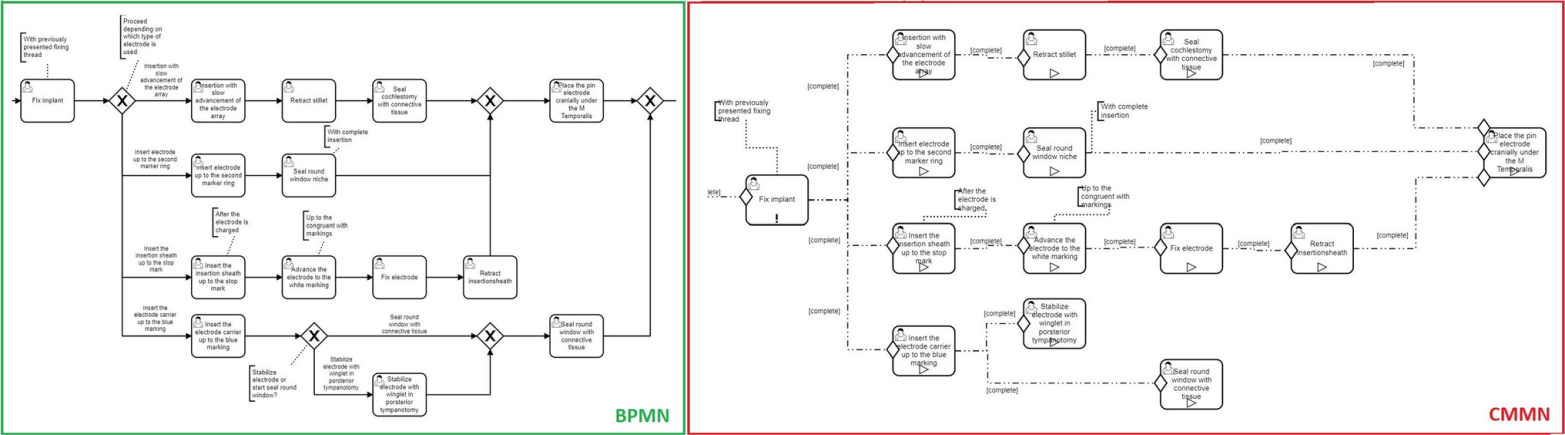
Excerpt of the CI use case contrasting variable process parts modeled in BPMN (left) and CMMN (right), including different procedures and optional steps

By modeling different procedures in the CI use case, the *XOR Gateway* in BPMN clarifies that only one path is possible (see Fig. 5, left). In CMMN, the same representation as for variable order is used but the paths are modeled as optional. Thereby, it cannot be differentiated if all paths or just one can be done (see Fig. 5, right). More dependency elements like *Stages* can counteract such uncertainties.

**Fig. 6:**
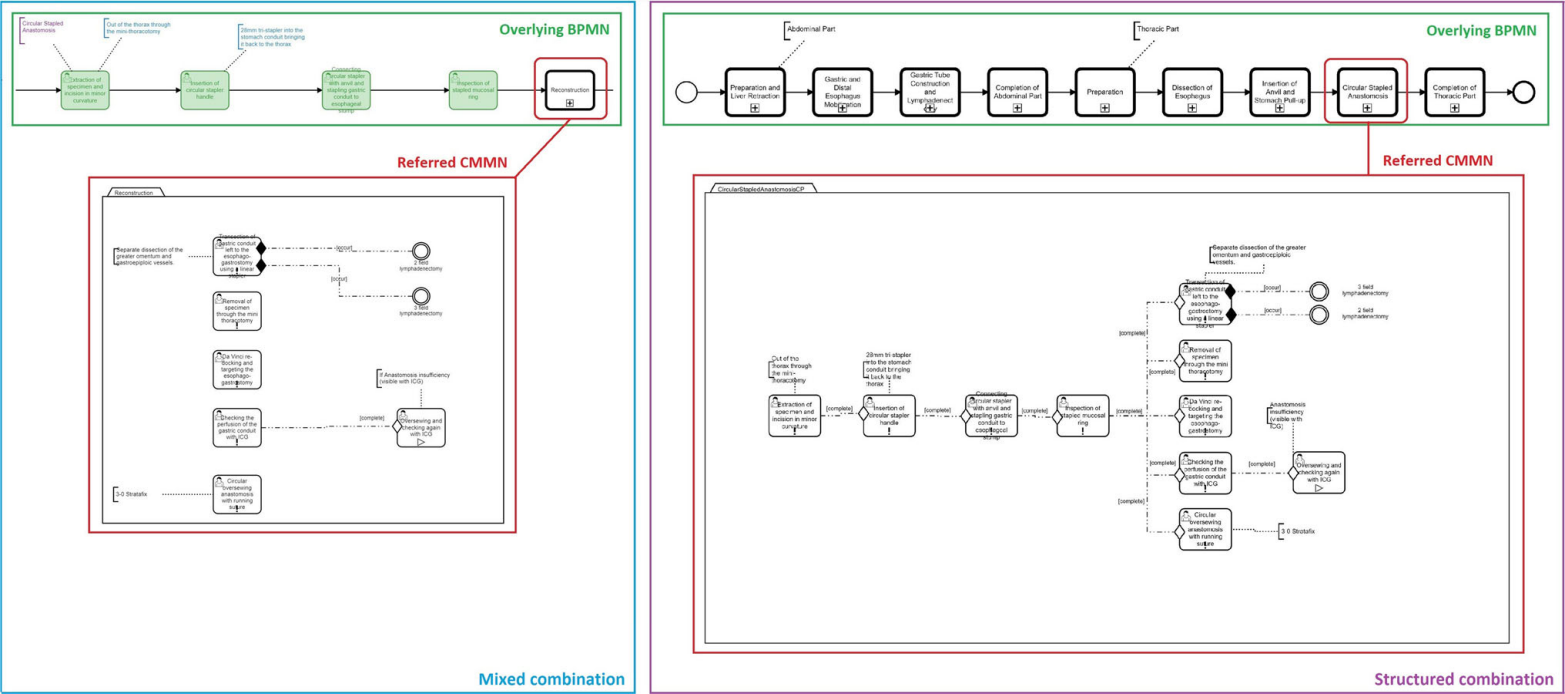
Excerpt of the RAMIE use case contrasting variable process parts modeled with an overlying BPMN and referred CMMN in a mixed (left) and structured (right) way

BPMN and CMMN can be combined in different ways. Within the mixed combination model, variable parts of the process are integrated in BPMN via *Call Activities*, referring to the sub process modeled in CMMN (see Fig. 6, left). As the back and forth between BPMN and CMMN is not consistent, no clear separation is apparent. The approach of a structured combination model uses an overlying BPMN comprised of the interventional phases and contains *Call Activities* to the cases (see Fig. 6, right), making it more clear as no mixture of *User Tasks* in BPMN and *Human Tasks* of CMMN are present. The modeled steps in BPMN and CMMN reflect the pure models.

Within the user tests, a total of 12 assessments could be made, seven for RAMIE and five for CI. Despite that a few comprehensibility problems occurred during observation, each test person was able to comprehend the models after getting into the peculiarities of the notations. Every test person stated that knowledge about the intervention is helpful to better understand the process models. In three observations, the test participant already had a few experiences with BPMN (beginner). The results are summarized in Tables 2 and 3.

**Table 2:** Overview of the results of the user tests for RAMIE process models, separated if the test person is familiar (fam.) or unfamiliar (unfam.) with the intervention, positive and negative remarks are depicted as ±

	RAMIE (Total of seven test persons)							
	BPMN		CMMN		Mixed combination		Structured combination	
	Fam	Unfam	Fam	Unfam	Fam	Unfam	Fam	Unfam
Amount of test persons	4 (two with beginner experience)	3	4	3	4	3	4	3
Average value of understanding (1 = very easy, 5 = very hard)	2 (2.5 without bias)	2.33	2.25	2.83	2.88	2.5	2.38	2.16
Number of ratings as the favorite model	1 (0 without bias)	2	1	0	0	1	1	1
Comprehension problems	Optional steps in variable order Join gateways Missing conditions for decisions		Optional steps and order Arrangement of the steps Paths		–		–	
Remarks	+ Good representation + No misunderstandings + Self-explanatory and sleek + Clear arrows to follow − More complex parts exhausting to read − Nested for parts with variability and optionality − More difficult to understand than CMMN		+ Advantages visible + More accurate depiction + All contents are directly visible and structure is given via stages + Obligatory and optional tasks more clear and easier than in BPMN + Variability in combination with obligatory and optional more clear than in BPMN − The main path is lost from sight − Need longer to get into the notation − More background knowledge needed − Events are inconsistent		+ Get the best of both notations − References are unattractive as they can be overlooked and logic changes		+ Good overview of the basic sequence + Referenced CMMNs remain small and clear + Complexity is depicted in detail − Subordinate structure less useful − Annoying to jump into the sub processes

**Table 3:** Overview of the results of the user tests for CI process models, separated if the test person is familiar (fam.) or unfamiliar (unfam.) with the intervention, positive and negative remarks are depicted as ±

	CI (total of five test persons)							
	BPMN		CMMN		Mixed combination		Structured combination	
	Fam	Unfam	Fam	Unfam	Fam	Unfam	Fam	Unfam
Amount of test persons	3	2 (one with beginner experience)	3	2	3	2	3	2
Average value of understanding (1 = very easy, 5 = very hard)	1.66	2 (3 without bias)	3.16	2.25	2.5	3	2.16	3
Number of ratings as the favorite model	3	1 (0 without bias)	0	1	1	0	1	1
Comprehension problems	Distinguish between gateways		Optional steps and paths, e.g., when one path is mandatory Variable order Next task after optional tasks (missing sequence)		–		–	
Remarks	+ Intuitive + Self-explanatory and logical + Elements and symbols easy to understand + Decisions depicted elegantly + Optional tasks are more clear than in CMMN − Variable order not depicted well − Gateways not intuitive − Good for linear processes but the reality is not linear		+ Not much harder to understand than BPMN + Variable tasks are much clearer than in BPMN + Events are better than in BPMN + “Complete” is nice as feedback + Complex parts better comprehensible due to symbols than BPMN − More complex notation (partly only at first glance) than BPMN − Less intuitive than BPMN, e.g., optional steps − Too much going on you need to pay attention to − Events less clear due to missing elements compared to BPMN		+ Steps are depicted very well in BPMN but variable parts can be modeled via CMMN − Structure less clear than for the structured combination − Level of detail not clear for references − Need to learn both notations		+ Good overview and structuring + Less nested than the mixed combination − Need to learn both notations	

### System tests (execution and control)

#### 2G BPMN

The RAMIE and CI test cases with simulated sensor data in reasonable order ran without errors. Steps in incorrect order were recognized by the system logic as not reasonable due to the inclusion of process knowledge. All required tasks need to be done in a valid order, variable tasks can be executed in any order and different paths be followed as modeled. Skipping or regressing tasks was not possible, except optional tasks can be skipped.

Semi-parallel tasks (see Fig. 4, left) are activated and present in the Camunda Tasklist after completing the task before (see Fig. 2). The token automatically jumps to all the tasks, making them possible to complete. Optional tasks after an *AND Gateway* are automatically skipped by the Camunda Engine if another semi-parallel task is recognized first. The optional task can be done afterward but this will not be reflected by Camunda. This peculiarity does not influence the recognition results but only the representation in Camunda. A *Sub Process* is automatically completed by Camunda after achieving the respective end event and started as the process token jumps to its first task.

#### 2G CMMN

The RAMIE and CI test cases with simulated sensor data in reasonable order also ran without errors. Not plausible tasks were recognized the same as for the BPMN, except first stage tasks. All required tasks need to be executed in a valid order, as skipping or regressing tasks was not possible. Variable tasks can be done as liked and optional tasks do not need to be done. Different paths could be executed as modeled. Limits were evident for delayed optional tasks and multiple paths (see subsection Discussion).

For tasks in variable order (see Fig. 4, right), all are activated and present in the Camunda Tasklist after completing the task or stage before, except for optional tasks. In contrast to the BPMN model, optional tasks can be still activated after another variable task was recognized. A *Stage* is not automatically completed by the engine if optional tasks are present within it and therefore needs to be completed “manually” by the system’s logic via the Camunda REST API. In contrast, the next *Stage* is activated automatically by Camunda after the completion of the one before. The case itself also needs to be completed and closed manually by the system after the last task.

#### 2G Structured combination

The results for the RAMIE and CI test cases were the same as for the CMMN models, except that in addition to stage changes case changes are made. The token of the process automatically jumps to the respective *Call Activities* in the BPMN model, after the process was started or the referenced case was closed. Furthermore, the cases need to be completed and closed manually via the Camunda REST API by the system’s logic.

## Discussion

### Modeling, execution, and control of surgical process models

#### Modeling

Modeling variable surgical processes via BPMN can lead to extensive models or workaround solutions, i.e., not modeled properly. In CMMN, the order of tasks does not have to be specified explicitly, while the models stay readable and comprehensible, as also stated in [18]. Furthermore, mandatory and optional steps can be easily differentiated using attributes instead of gateways. Tasks that occur only once or multiple times [18] and different procedures [21] can be depicted, whereas a BPMN model would become too complex and incomprehensible. However, clear paths can be depicted in BPMN, whereas CMMN does not provide this information to the same extent, as also stated in [17]. More background knowledge of the process is needed [17] to comprehend if the tasks have a variable order or if only one path is possible, as in CMMN it is more difficult to represent dependencies. By combining BPMN with CMMN, the strengths of both notations can be combined, either by mixing structured parts with unstructured parts or referring from a structured overlying BPMN to unstructured CMMNs.

The user tests showed for RAMIE and CI process models that impressions and opinions differ extremely. BPMN was mostly described as intuitive, clear, and self-explanatory but more complex parts led to comprehensibility problems. CMMN was rather stated as less intuitive and more complex, especially the depiction of order and optional tasks. On the other hand, variable, required, and optional tasks were more clear. For the combination models, some highlighted that using both notations and dividing complexity in sub processes is advantageous, while others disliked this mixture and the references. Despite these remarks, all models scored a similar average between 2 and 3 (easy to neutral), expect two outliers in the CI models. Being familiar with the intervention was stated helpful but differences are not visible in the results. Also, the rating of the favorite model is distributed. For RAMIE, the BPMN and structured combination are slightly favored, a more clear tendency to BPMN was spotted for CI. According to the users, BPMN seems to be the favorite and best to comprehend, nevertheless, also the notation of CMMN scored in some aspects. Although in [18] and in our tests CMMN was mentioned to be more difficult to comprehend, the average scores we obtained indicate comparable comprehensibility. Furthermore, we assume that each approach can be made likewise comprehensible through explanation and practice, as a learning curve was visible during observation.

#### Execution and control

In the context of situation recognition, the validity of the SPMs was demonstrated. The execution and control of the selected models were challenging. For BPMN, only a path needs to be set for optional tasks and tasks to be completed. For CMMN, apart from completing tasks, optional tasks are to be started manually and *Stages* are to be completed via the system using the Camunda REST API. To control the structured combination model, a similar effort is needed for CMMN only models. In the case of a mixed combination model, more hopping between BPMN and CMMN control would be needed, making execution and control more complex.

The XML structure of the models is also different. In BPMN, the path can be clearly followed via the *Sequence Flows* to determine tasks. In CMMN, elements are structured within *Plan Items* which contain the relevant information about the assigned *Stage* or *Human Task* as well as *Entry Criteria* and rules. As CMMN is less structured, it lacks sequences, branches, and start and end of *Stages*, so the order can be chosen more freely [17], enabling the variable execution of a task. However, this led to a higher error-proneness in validating stage/case changes and when differentiating between variable order, different paths, and optional tasks, so that falsely recognized delayed optional tasks and the execution of multiple exclusive paths were possible. This is due to the chosen modeling approach with CMMN. For example, different paths are depicted as optional although one path needs to be done. Such peculiarities might be solved via other CMMN approaches or an extension of the control logic that is aware of such exceptions. Up to now, the CMMN process control does not have the same deepness as for BPMN to filter out false positives. As CMMN relies on the user or the system knowing what is reasonable and provides all options, integrating more data like similar, executed processes to gain better knowledge about next possible steps is crucial. Overall, it was more difficult to read out the needed information of CMMN and there still is a lack of including all conditions and special cases. Although DMN was excluded in this work, it might be a possible supplement for better workflow control (see [18]) and may counteract the limitations mentioned above.

### Applicability and transferability

Integrating CMMN into the system architecture was a crucial step for the applicability and transferability of the SRS. The results show that the SRS not only can support BPMN models of standardized interventions but also complex interventions in both notations, making it applicable for various kinds of interventions and circumstances. Furthermore, combining BPMN and CMMN by referring to CMMN cases based on an overall BPMN structure allows for adaptivity, as addressed in [19], enabling interchangeable cases. For the same intervention conducted in different hospitals, hospital-specific CMMN cases can be used within the BPMN process. On the other side, all variants of the intervention can be modeled within one case [21]. Moreover, process parts outsourced as CMMN cases can be used for multiple interventions. Another aspect to consider is the changeability and extendability of the process models. This could lead to a major change for a more structured model, whereas a more straightforward integration is expected for a less structured model. By supporting CMMN and combination models, the SRS became more transferable as interventions can be supported in a new and more variable way.

Using models for several procedures, interventions, and hospitals can lead to even more complex models, not always just containing the possible steps for the specific use case that it is used for. To enable that the model is still applicable to the different circumstances, the SRS needs to have enough knowledge about the most probable next steps. Recordings of past processes are then more than necessary to be included in the situation recognition workflow to enable error tolerance against unplausible situations. With this concept, the applicability and transferability of the SPMs, and therefore of the SRS, can be further optimized.

### Final remarks

Concluding, BPMN is more convenient for very strict courses but CMMN convinces in its flexibility and comprehensibility to be a good alternative for modeling variable surgical processes, especially when BPMN models would become too complex or workarounds should be avoided. We derive, also referring to the results of [17, 18, 21], that CMMN enables a clear representation of highly variable processes in surgery although non-optimal representations need to be reconsidered which led to false positives. A combination of BPMN and CMMN could also be reasonable, as equally stated by [17, 18], and was rated similarly in the user tests. The control of CMMN and combination models in the Camunda Engine offers added value to the SRS, although more effort and knowledge are needed for execution and control to be integrated into the system’s logic. As the control logic is crucial to filter out false positive recognitions, it needs to be extended to reduce limitations occurring due to the variable models and to exploit the full potential of CMMN.

Overall, BPMN and CMMN both have their advantages and disadvantages. The proper modeling notation needs to be chosen depending on the use case. For a more standardized process, BPMN is more suitable, for more variable process parts, CMMN can be a good alternative to depict more adequately in less complexity. Nevertheless, the modeling approach for some CMMN parts should be reconsidered in means of either modeling more restrictive or depicting more loosely to better reflect reality. CMMN and combination models can especially be suitable for transferability among procedures, interventions, and hospitals, and should be considered for such goals.

## Conclusion

As BPMN may become quite complex for surgical interventions, CMMN is an alternative addressing the variability of weakly structured processes like complex, non-standardized surgical interventions. This work showed that, compared to BPMN, CMMN allows the flexibility needed to correctly depict variable, intra-operative processes to be used within a situation recognition for execution and control. Therefore, CMMN is suitable to be used in addition to BPMN for flexible process parts (a combination of BPMN and CMMN) or as a replacement for the entire process (CMMN only). Especially a combination of both notations promises to optimize the interchangeability and transferability of the models. Nevertheless, the used modeling notation depends on the use case, knowledge about the techniques, and own preferences. A clear recommendation can therefore not be given.

Overall, the support of BPMN, CMMN, and combination models including the integration of process knowledge optimizes the applicability and transferability of the SRS. As demonstrated in the RAMIE and CI use case, complex processes can be depicted. To further evaluate the potential of CMMN models to execute and control surgical interventions in the context of situation recognition, more interventions should be modeled using CMMN or a combination of BPMN and CMMN using different approaches to depict variants and dependencies.
